# The role of the right prefrontal cortex in the retrieval of weak representations

**DOI:** 10.1038/s41598-022-08493-6

**Published:** 2022-03-16

**Authors:** Kyongmyon Yi, Juyeon Heo, Jiyun Hong, Chobok Kim

**Affiliations:** Department of Psychology, Kyungpook National University, Daegu, 41566 South Korea

**Keywords:** Human behaviour, Psychology, Cognitive neuroscience

## Abstract

Although recent studies have shown the importance of control in creative problem solving, the neural mechanisms of control processes engaged in retrieval of weak representations, which is closely linked to creative problem solving, remain unclear. The current study aimed to examine the neural mechanisms associated with retrieval of weak representations using functional magnetic resonance imaging and their potential relationships with creativity task performance. For this purpose, participants performed an experimental task that enabled us to directly compare between retrieval of previously unattended-and-weak representations and attended-and-strong representations. Imaging results indicated that the right anterior dorsolateral prefrontal cortex (aDLPFC) was selectively engaged in retrieval of weak representations. Moreover, the right aDLPFC activations were positively correlated with individuals’ creativity task performance but independent of attention-demanding task performance. We therefore suggest that the right aDLPFC plays a key role in retrieval of weak representations and may support creative problem solving.

## Introduction

Previous studies have emphasized the contribution of linking between weakly-associated representations in memory to generate creative ideas for solving problems^1–3^. According to a traditional account of creative processes^3^, generating creative ideas can be best understood as a spreading activation of memory representations that are interconnected within a distributed semantic network of long-term memory (LTM), suggesting that retrieving one concept in memory diffusely activates other connected concepts^3^. Accordingly, this phenomenon would explain the passive retrieval of weakly-associated representations in memory. For example, strongly-associated representations for a given concept (e.g., “candle” and “flame”) are immediately retrieved into working memory (WM), whereas weakly-associated representations (e.g., “candle” and “halo”) become more slowly available in WM^4^.

However, accumulative evidence suggests the importance of top-down control in creative idea generation^5–8^. For example, behavioral studies demonstrated that individuals with higher control abilities can generate creative responses earlier than those with lower abilities^7–9^. Further, neuroimaging studies reported neural correlates in frontal regions, including the inferior frontal gyrus (IFG) and frontopolar cortex, suggesting that these regions are associated with controlled semantic retrieval^10^, inhibition of strongly-associated representations^11,12^, or semantic processing of weakly-associated representations^13,14^. In addition, the dorsolateral prefrontal cortex (DLPFC) and dorsal anterior cingulate cortex (dACC) are thought to be engaged in WM functions during creativity tasks, such as active maintenance and monitoring of goal-related representations^15–18^.

Although several brain regions have been suggested to be involved in control processes during creative idea generation, the underlying neural mechanisms of control processes involved in retrieving weakly-associated representations still remains unclear. Indeed, identifying direct evidence of these mechanisms remains challenging in an experimental setting. This difficulty arises given that a direct comparison between the control processes engaged in retrieving weakly- and strongly-associated representations to WM requires the prerequisite that participants retrieve the given representations from their semantic memory with the same representational strength for both weak and strong associations. However, the strength for any given representations is largely obscured across individuals^19^ because semantic networks in their LTM substantially vary with their own experience and knowledge^20^, which imposes constraints on experimental manipulation of representations in LTM.

One promising method that may overcome this challenge involves assessing how weak representations (i.e., those with low representational strength) are retrieved from WM, rather than how weakly-associated representations are retrieved from LTM. One rationale is that the retrieval operations that act upon WM and LTM share common processes^21–23^, as suggested by the state-based WM models^24–26^. Another rationale is that the activation levels of WM representations can be determined by attentional modulation during the encoding processes: (1) Focused items relative to unfocused items remain strengthened in WM, thereby remaining easily accessible^27^ and (2) Neural responses are greater for focused items compared to ignored items^28,29^. In this context, the representational strength can be manipulated according to whether the stimuli being retrieved into WM were previously related to focused or ignored items. Accordingly, it is possible to investigate neural mechanisms of the control processes engaged in the retrieval of weak representations (“retrieval of weak representations” or RWR) with low representational strength, compared to strong representations with high representational strength.

**Figure 1:**
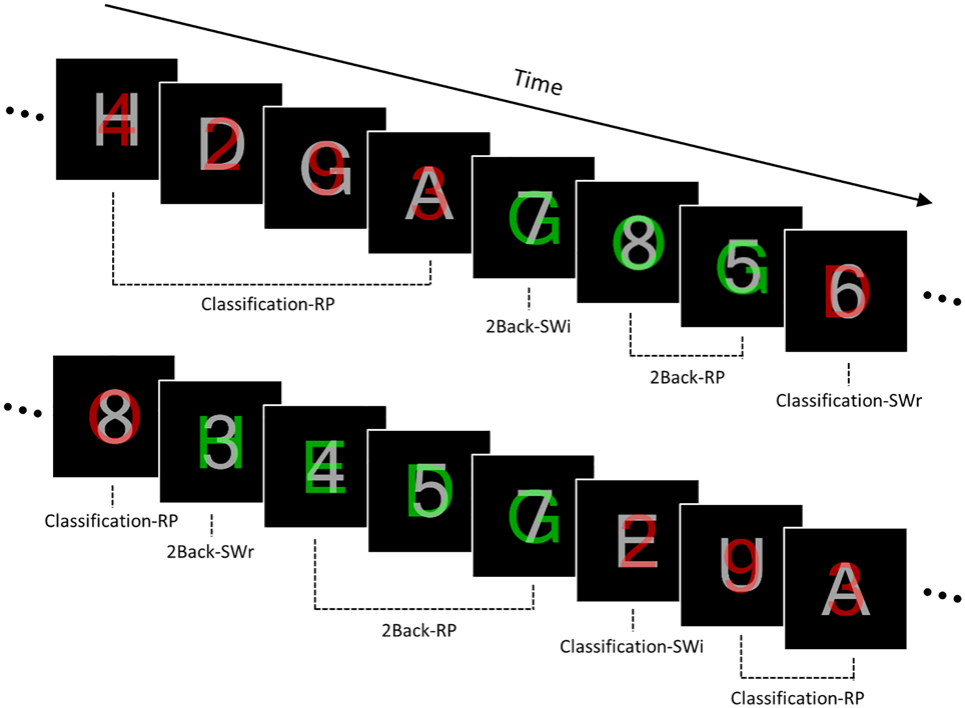
Task stimuli and conditions used in the experiment. For red stimuli, the task required participants to classify the stimuli as odd/even or vowel/consonant according to whether the current red target was a number or letter, respectively (i.e., the classification task). When the stimulus color was changed to green, participants were required to determine whether the current target in green was identical to the stimulus that was presented in red or in gray two trials before. *RP* repeat, *SWr* switch-relevant, *SWi* switch-irrelevant.

In the present study, we aimed to reveal the neural mechanisms of control processes underlying the retrieval of weak representations. To this end, we conducted a functional magnetic resonance imaging (fMRI) experiment to compare the retrieval processes for previously ignored stimuli (i.e., retrieval of weak representations, or RWR) with those of previously attended stimuli (i.e., retrieval of strong representations). To manipulate the weak or strong representations to be retrieved into WM, we designed a task paradigm including a 2-back updating task (2Back), which appeared after a classification task (Classification), with numbers or letters: retrieval of a strong representation involved presenting a number (or a letter) during the 2Back trials after performing consecutive number (or letter) Classification trials; retrieval of a weak representation involved presenting a number (or a letter) during the 2Back trials after performing sequential letter (or number) Classification trials (Fig. 1). Importantly, the number and letter stimuli were superimposed onto each other during the Classification trials in order to ensure that focusing on a number or letter stimulus resulted in ignoring the other.

Additionally, we administered the Alternate Uses Test (AUT) as well as the flanker and response switch tasks to identify whether participants’ neural responses during RWR are related to their behavioral performance in a creativity task and/or attention-demanding tasks. However, it should be noted that these brain–behavior correlation analyses were nonindependent (i.e., neural responses resulted from significantly activated regions in voxel-wise analyses)^30,31^.

Finally, we took several factors into consideration to examine RWR-specific neural mechanisms within an experimental setting. First, since the task requires switching from external stimuli during the Classification trials to internal representations, which were presented two trials earlier regardless of their intensities, and then comparing the internal representations with the current stimulus in the current 2Back trial, brain activations related to switching from external stimuli to internal representations must be differentiated from RWR-related activations. Second, it is important to exclude any activation associated with consecutive repeating 2Back trials. With this experimental design and criteria, we examined the brain regions specifically associated with the processing of previously ignored representations into WM, i.e., RWR. We expected that cortical regions associated with RWR would be subregions of the lateral prefrontal cortex, which are known to be responsible for attentional control^32,33^.

## Results

### Behavioral results

The experimental design was a two-way within-subject design. The first factor (Task) was composed of 2-back updating task (2Back) and classification task (Classification) conditions. The second factor (Switch-type) consisted of three conditions: trials that were required to switch from one task to the other and the stimulus domain was previously task-relevant (SWr) or previously task-irrelevant (SWi), and trials that were repeated within the same task and stimulus domain as the previous trial (RP) (for details, see “Materials and procedures”).

**Figure 2:**
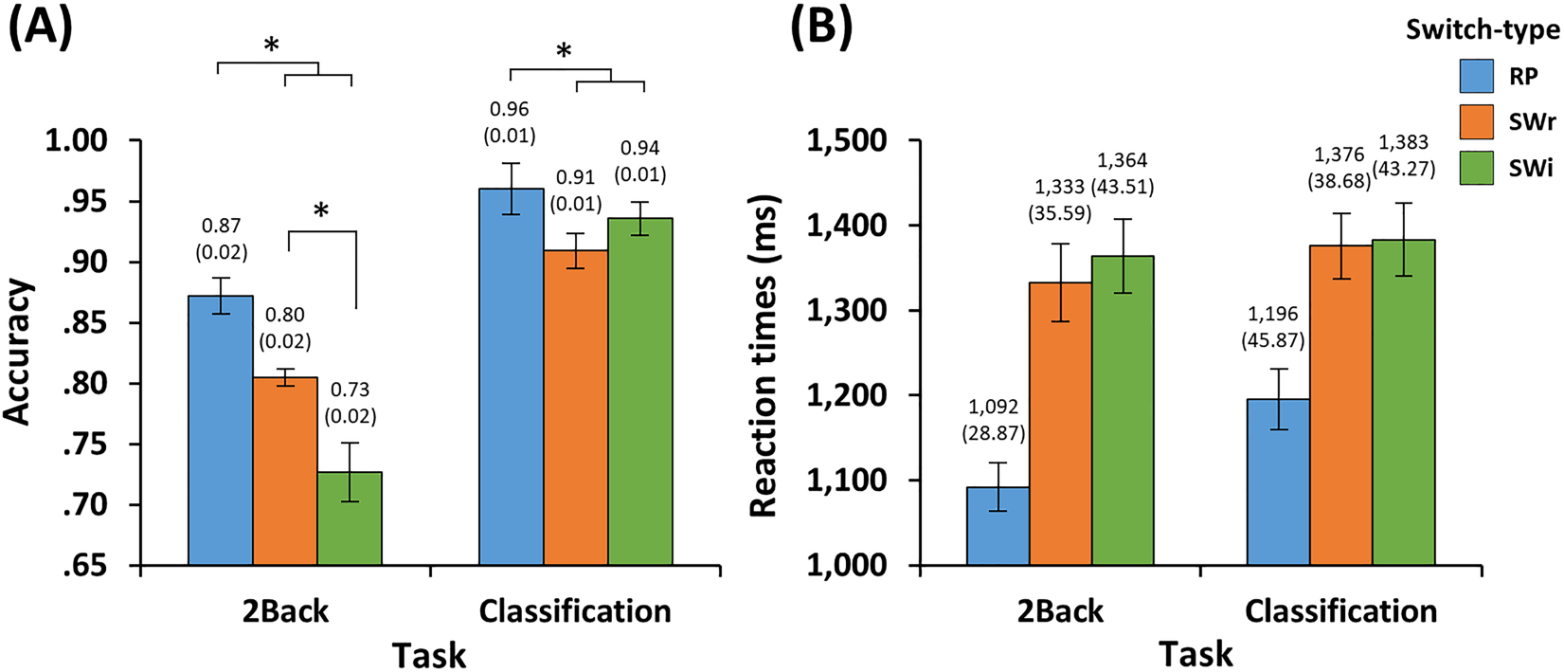
Behavioral results. Mean (**A**) accuracy and (**B**) reaction times for each experimental condition. The *post-hoc* pairwise comparisons were tested using paired simples t-tests based on significant interactions. Error bars indicate the standard error of the means. *RP* repeat, *SWr* switch-relevant, *SWi* switch-irrelevant.

Mean accuracy and RTs were analyzed in the context of a 2 (Task: 2Back and Classification) × 3 (Switch-type: SWr, SWi, and RP) repeated-measures ANOVA. As illustrated in Fig. 2A, for accuracy, the main effect of Task was significant [F(1,29) = 130.283, *p* < 0.05, ${\eta }_{p}^{2}$ = 0.818] due to higher accuracy in Classification than 2Back. The main effect of Switch-type was also significant [F(2,58) = 28.255, *p* < 0.05, ${\eta }_{p}^{2}$ = 0.493] due to the accuracy of RP being higher than that of both SWr [*p* < 0.05, 95% CI (0.032, 0.085)] and SWi [*p* < 0.05, 95% CI (0.055, 0.116)] with no significant difference observed between SWr and SWi [*p* = 0.120, 95% CI (− 0.005, 0.059)]. In addition, the interaction between Task and Switch-type was significant [F(2,58) = 10.548, *p* < 0.05, ${\eta }_{p}^{2}$ = 0.267] due to the accuracy of 2Back-RP being higher than that of 2Back-SWr [t(29) = 3.696, *p* < 0.05, Cohen’s *d* (*d*) = 0.670, 95% CI (0.030, 0.105)] while that of 2Back-SWr was higher than that of 2Back-SWi [t(29) = 3.007, *p* < 0.05, *d* = 0.642, 95% CI (0.025, 0.133)]; however, the accuracy of Classification-RP was higher than that of both Classification-SWr [t(29) = 4.472, *p* < 0.05, *d* = 0.800, 95% CI (0.027, 0.072)] and Classification-SWi [t(29) = 2.391, *p* < 0.05, *d* = 0.406, 95% CI (0.004, 0.045)] with no significant difference observed between Classification-SWr and Classification-SWi [t(29) = − 1.900, *p* = 0.067, *d* = 0.328, 95% CI (− 0.053, 0.002)].

As shown in Fig. 2B, RT analysis indicated that the main effect of Switch-type was significant [F(2,58) = 42.893, *p* < 0.05, ${\eta }_{p}^{2}$ = 0.597], which is attributable to faster RTs for RP than those of SWr [*p* < 0.05, 95% CI (− 288, − 133)] and SWi [*p* < 0.05, 95% CI (− 310, − 150)] with no significant difference observed between SWr and SWi [*p* = 0.911, 95% CI (− 67, 28)]. However, the main effect of Task [F(1,29) = 3.210, *p* = 0.084, ${\eta }_{p}^{2}$ = 0.100, 95% CI (− 7, 118)] and the interaction between Task and Switch-type were not significant [F(2,58) = 3.055, *p* = 0.055, ${\eta }_{p}^{2}$ = 0.095].

Given that the participants were native Korean speakers, there was a possibility that performances between tasks with letters (i.e., those with English consonants and vowels) and numbers would differ. To address this possibility, the mean accuracies and RTs between number and letter trials in 2Back and Classification conditions were analyzed using paired samples t-tests. Results showed that there were no significant differences in accuracy between the number (M = 0.856, SD = 0.078) and letter trials (M = 0.860, SD = 0.071) [t(29) = − 0.564, *p* = 0.577, *d* = 0.063, 95% CI (− 0.021, 0.012)] nor in RTs between the number (M = 1,248 ms, SD = 175) and letter trials (M = 1,255 ms, SD = 163) [t(29) = − 0.475, *p* = 0.638, *d* = 0.042, 95% CI (− 38, 24)]. These results show that the usage of English letters in our task did not cause problems to native Korean speaking participants.

### Imaging results

**Figure 3:**
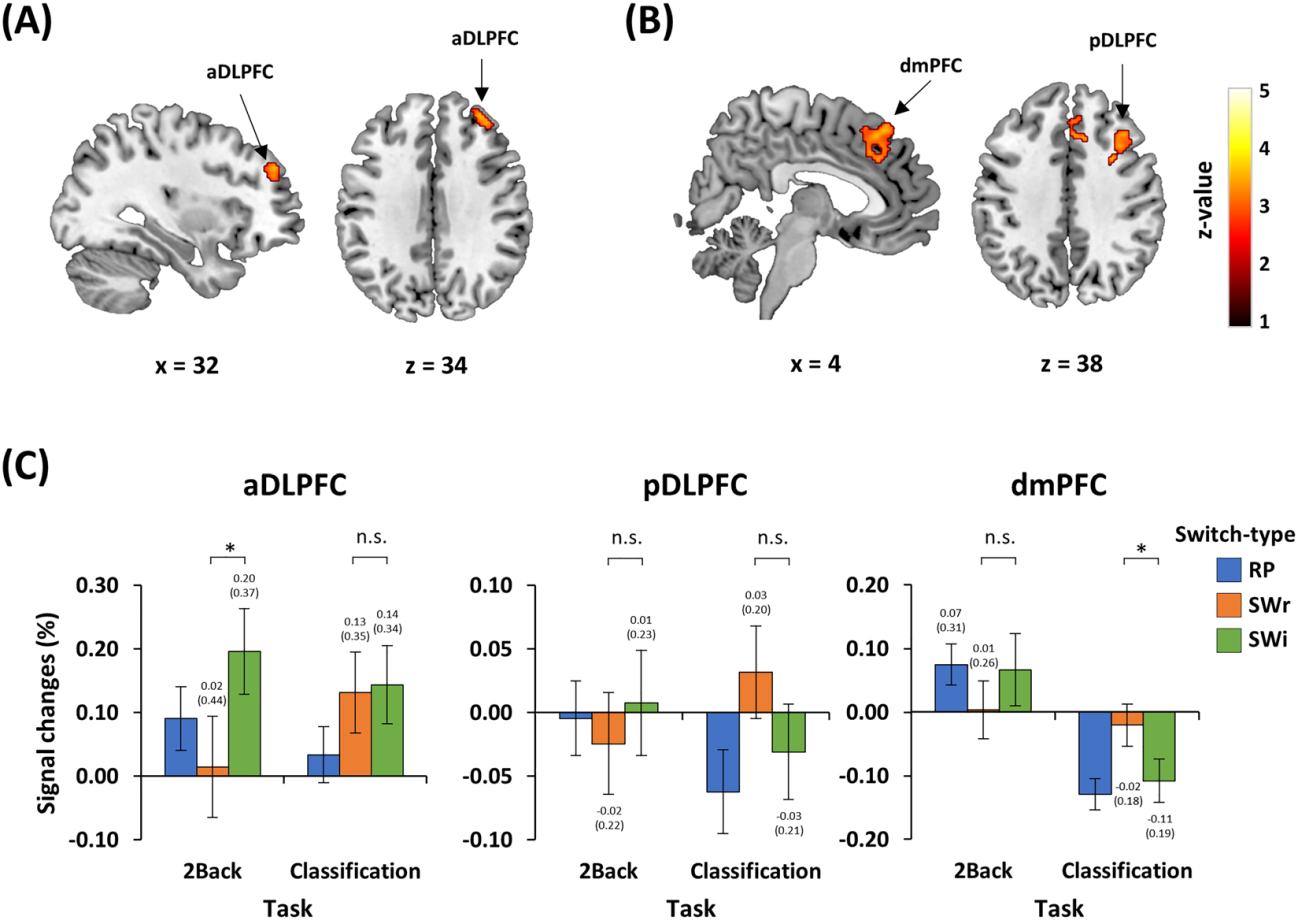
Significant brain activations associated with the retrieval of weak representations (RWR) via 2 × 2 interaction [(2Back-SWi–2Back-SWr) > (Classification-SWi–Classification-SWr)] and pairwise comparisons of the signal change in the identified clusters. (**A**) Activation of the right anterior dorsolateral prefrontal cortex (aDLPFC) was uniquely associated with RWR interaction following exclusive masking of the simple updating and switching. (**B**) Activation of the posterior dorsolateral prefrontal cortex (pDLPFC) and dorsomedial prefrontal cortex (dmPFC) mainly overlapped with regions activated by simple updating. (**C**) The bar graph represents the signal changes of the aDLPFC, pDLPFC, and dmPFC for each experimental condition. The *post-hoc* pairwise comparisons were tested using paired simples t-tests based on significant interactions. Error bars indicate the standard error of the means. The statistical threshold maps were shown at *p* < 0.05 cluster-level correction for multiple comparisons with the cluster size defined at a voxel level, uncorrected *p* < 0.001. Color bars represent the t-values. *RP* repeat, *SWr* switch-relevant, *SWi* switch-irrelevant. The activated clusters are overlapped onto the ch2better.nii template using mricron software (version 09.02.2019, https://www.nitrc.org/projects/mricron)^71^.

Functional imaging data were first examined to identify RWR-related brain regions via the interaction contrast [i.e., (2Back-SWi–2Back-SWr) > (Classification-SWi–Classification-SWr)] in the context of whole-brain analysis. Initial results showed significant activations in distinct clusters in the frontal area including the right anterior dorsolateral prefrontal cortex (aDLPFC) (BA 46), posterior dorsolateral prefrontal cortex (pDLPFC) (BA 46), and dorsomedial prefrontal cortex (dmPFC) (BA 8/32) (Table 1 and Fig. 3A,B). To verify RWR-specific cortical regions among the aforementioned frontal regions, we applied exclusive masking with the simple effects of both WM updating (i.e., 2Back-RP > Classification-RP) (Supplementary Fig. S1A) and switching (i.e., 2Back-SW > 2Back-RP) (Table 2 and Supplementary Fig. S1B). This verification indicated that RWR-specific activation was only found in the right aDLPFC, whereas the right pDLPFC and dmPFC activations overlapped with the masking, particularly with simple updating regions.

**Table 1:** Significant areas of retrieval of weak representations (RWR).

Region	BA	L/R	Cluster size	MNI coordinate			z-value
				X	Y	Z	
**RWR contrast (2Back-SWi–2Back-SWr > Classification-SWi—Classification-SWr)**							
aDLPFC	46	R	126	32	46	34	4.23
pDLPFC	46	R	288	32	22	34	4.48
dmPFC	8/32	R	369	2	30	48	4.20
**RWR contrast (2Back-SWi–2Back-SWr > Classification-SWi–Classification-SWr) with exclusive masking**							
aDLPFC	46	R	121	32	46	34	4.23

All z-scores listed above were found at FDR-corrected p < 0.05 with the cluster size defined at a voxel level, uncorrected p < 0.001.

*BA* Brodmann area, *L* left, *R* right, *a* anterior, *p* posterior, *DLPFC* dorsolateral prefrontal cortex, *dmPFC* dorsomedial prefrontal cortex, *SWr* switch-relevant, *SWi* switch-irrelevant.

**Table 2:** Significant areas of activation for simple updating and switching.

Region	BA	L/R	Cluster size	MNI coordinate			z-value
				X	Y	Z	
**Simple updating (2Back-RP > Classification-RP)**							
Frontopolar/ventrolateral cortex	10/47	L	325	− 38	44	2	5.20
	10/47	R	244	42	50	− 4	4.85
IFG	44	L	80	− 50	14	10	4.17
MFG	9/46	L	1152	− 40	12	54	5.45
IFG/MFG	9/44/46	R	2389	52	16	16	5.47
dmPFC/ACC	8/32	L/R	1243	2	34	40	6.18
Anterior insula		L	201	− 32	20	− 4	5.59
		R	260	32	26	− 4	5.49
IPL/angular gyrus	7/40	L	736	− 50	− 46	44	4.60
	7/40	R	1500	48	− 42	52	5.67
**Simple switching (2Back-SW > 2Back-RP)**							
MFG	45	L	156	− 40	36	20	4.27
PM	6	R	577	24	8	58	4.63
dmPFC/ACC/IFG/MFG/PM	8/32/6/9/44/45	L	2745	− 6	20	48	5.34
IPL	40	L	746	− 46	− 30	40	5.18
Angular gyrus	7/40	L	159	− 26	− 60	38	4.09
	7/40	R	104	36	− 68	38	4.13
Precuneus	7	L	929	− 8	− 70	54	5.52
Calcarine gyrus	19	R	83	20	− 72	8	4.31

All z-scores listed above were found at FDR-corrected p < 0.05 with the cluster size defined at a voxel level, uncorrected p < 0.001.

*BA* Brodmann area, *L* left, *R* right, *IFG* inferior frontal gyrus, *MFG* middle frontal gyrus, *dmPFC* dorsomedial prefrontal cortex, *ACC* anterior cingulate cortex, *PM* premotor cortex, *IPL* inferior parietal lobule, *RP* repeat, *SW* updating switch.

Regions of interest (ROI) analyses were conducted to characterize activation patterns of the three ROIs (Fig. 3C). Percent signal changes (PSCs) extracted from these ROIs were compared between 2Back-SWi and 2Back-SWr, and between Classification-SWi and Classification-SWr. Among the regions, only the right aDLPFC showed RWR-specific activations. In detail, the PSCs of 2Back-SWi (M = 0.196, SD = 0.367) were higher than those of 2Back-SWr (M = 0.015, SD = 0.436) [t(29) = 2.363, *p* < 0.05, *d* = 0.448, 95% CI (0.024, 0.338)], while there was no difference between Classification-SWi (M = 0.144, SD = 0.335) and Classification-SWr (M = 0.131, SD = 0.347) [t(29) = 0.228, *p* = 0.821, *d* = 0.036, 95% CI (− 0.098, 0.123)]. In addition, the PSCs of 2Back-SWi were marginally higher than those of 2Back-RP (M = 0.091, SD = 0.274) but not at a significant level [t(29) = 1.788, *p* = 0.084, *d* = 0.324, 95% CI (− 0.015, 0.225)]. The pDLPFC showed no difference between 2Back-SWr (M = − 0.024, SD = 0.219) and 2Back-SWi (M = 0.007, SD = 0.227) [t(29) = − 0.970, *p* = 0.340, *d* = 0.150, 95% CI (− 0.099, 0.035)], whereas Classification-SWr (M = 0.032, SD = 0.199) and Classification-SWi (M = − 0.031 SD = 0.205) were marginally but not significantly different [t(29) = 1.721, *p* = 0.096, *d* = 0.313, 95% CI (− 0.012, 0.137)]. Similarly, for the dmPFC, the PSCs of Classification-SWr (M = − 0.020, SD = 0.182) were higher than those of Classification-SWi (M = − 0.108, SD = 0.185) [t(29) = 2.979, *p* < 0.05, *d* = 0.482, 95% CI (0.027, 0.148)], but there was no difference between 2Back-SWr (M = 0.004, SD = 0.251) and 2Back-SWi (M = 0.067, SD = 0.310) [t(29) = − 1.467, *p* = 0.153, *d* = 0.221, 95% CI (− 0.151, 0.025)]. Overall, these results demonstrate that right aDLPFC activation was closely associated with RWR, in accordance with the whole-brain results.

Next, based on the *post-hoc* ROI analyses, correlation analyses were conducted to identify whether the right aDLPFC activation associated with RWR was related to behavioral measurements in creativity task performance (AUT scores) and/or attentional-demanding task performance (the interference effect and switch cost). The results showed that the differences in PSCs between 2Back-SWi and 2Back-SWr was positively correlated with the total score [r = 0.475, corrected *p* < 0.05, 95% CI (0.139, 0.713)]. The results of AUT subscores (fluency, flexibility, and originality) showed that right aDLPFC activation was positively correlated with fluency [r = 0.446, corrected *p* < 0.05, 95% CI (0.102, 0.695)] and flexibility [r = 0.474, corrected *p* < 0.05, 95% CI (0.137, 0.713)], but unrelated to originality [r = 0.278, uncorrected *p* = 0.278, 95% CI (− 0.091, 0.580)]. Additionally, right aDLPFC activation was unrelated to the interference effect [r = 0.133, uncorrected *p* = 0.483, 95% CI (− 0.238, 0.470)] and switch cost [r = − 0.004, uncorrected *p* = 0.981, 95% CI (− 0.363, 0.356)].

Additionally, functional data were analyzed to identify regions associated with the 2 × 3 interaction [i.e., (2Back-SW–2Back-RP) > (Classification-SW–Classification-RP)] in the context of whole-brain analysis (despite this being beyond the scope of the current study). Results showed that only the presupplementary motor area (preSMA, BA 6) was activated (Supplementary Fig. S2A). Accordingly, PSCs were also extracted from this ROI and compared in a pair-wise manner between conditions (Supplementary Fig. S2B). Specifically, the PSCs of 2Back-SW (M = 0.319, SD = 0.219) were higher than those of 2Back-RP (M = 0.206, SD = 0.163) [t(29) = 3.821, *p* < 0.05, *d* = 0.582, 95% CI (0.052, 0.173)] while those of 2Back-SWi (M = 0.368, SD = 0.237) were higher than those of 2Back-SWr (M = 0.270, SD = 0.254) [t(29) = 2.461, *p* < 0.05, *d* = 0.405, 95% CI (0.016, 0.181)]; however, there was no difference between Classification-SW (M = 0.152, SD = 0.185) and Classification-RP (M = 0.208, SD = 0.166) [t(29) = − 1.978, *p* = 0.057, *d* = 0.323, 95% CI (− 0.113, 0.002)] nor between Classification-SWr (M = 0.151, SD = 0.183) and Classification-SWi (M = 0.153, SD = 0.218) [t(29) = − 0.078, *p* = 0.938, *d* = 0.010, 95% CI (− 0.061, 0.056)].

## Discussion

In the present study, we aimed to identify the neural mechanisms of control processes underlying the retrieval of weak representations in WM (i.e., RWR). Our fMRI results indicated that only the right aDLPFC was selectively engaged in RWR. Interestingly, correlation analyses showed that right aDLPFC activation was positively correlated with the participants’ creativity task performance, but that it was unrelated to attention-demanding task performance. Below, we discuss our novel findings with a focus on the functions of the right aDLPFC as well as their potential roles in creative problem solving.

Three frontal regions in the right hemisphere, the aDLPFC, pDLPFC, and dmPFC, were found to be related to the interaction contrast, but only the right aDLPFC was significantly associated with RWR by exclusion of regions independently related to WM updating and attentional switching. The activation of this region was greater when retrieving previously unattended-and-weak representations in WM than when retrieving previously attended-and-strong representations, which was similar in the activation level to regions related to switching of attention toward external stimuli (i.e., Classification-SWi and Classification-SWr). Furthermore, in our *post-hoc* correlation analyses, we observed that the right aDLPFC was the only region showing a positive relation between its activation and the creativity task performance. In contrast, activation of the aDLPFC was unrelated to the attention-demanding task performance, including the interference effect and switch cost. Given that the generation of more creative responses, i.e., higher creativity task performance, involves retrieval of remote-and-weak representations in the semantic network^34–36^, these *post-hoc* results may suggest that the magnitude of the control processes in retrieving weak representations in WM positively covaried with the creative level of the generated ideas. However, the results should be interpreted carefully because of a non-independent ROI selection (i.e., ROIs were selected from significant regions from the whole-brain analyses)^30,31^.

Consistent with our finding that the right aDLPFC is engaged in RWR, previous neuroimaging studies on creative problem solving, in which functional connectivity methods were applied, have suggested that this region is a core of control processes during creative idea generation^37,38^. For example, Beaty, et al.^37^ performed an fMRI study to identify the neural mechanisms associated with AUT, conducting a series of analyses, including multivariate pattern analysis, seed-to-voxel, and ROI-to-ROI functional connectivity analyses. They found that the right DLPFC (peak MNI: x = 36, y = 44, z = 20) was commonly involved in creative problem solving in interaction with other regions, including the default mode network. Similarly, Pinho, et al.^38^ found that the right DLPFC (peak MNI: x = 40, y = 42, z = 29) exhibited functional connectivity with diverse regions of the default mode network during musical improvisation. Importantly, these regions are very close to the region found in the current study (peak MNI: x = 32, y = 46, z = 34).

It is worth noting that our results indicate the right-lateralized DLPFC is associated with RWR despite the fact that our task used verbal and numerical stimuli. According to the novelty-routinization hypothesis of hemispheric specialization^39,40^, the right hemisphere is critical for processing novel situations while the left hemisphere is critical for processing established cognitive strategies and representations. Previous studies using neuroimaging and neuropsychological approaches have consistently reported the importance of the right-lateralized DLPFC in creative problem solving such as ill-structured problems^41,42^. In addition, there is evidence that transcranial direct current stimulation of the right DLPFC enhances creativity task performance^43^. Although these studies do not provide specific regions selectively associated with creative problem solving, they do support our findings.

According to their functional roles in control processes, the right aDLPFC has been distinguished from the pDLPFC. For instance, Cieslik, et al.^44^ suggested that the anterior portion of the right DLPFC (peak MNI: x = 30, y = 43, z = 23) is closely related to attention processes, whereas the posterior portion (peak MNI: x = 37, y = 33, z = 32) is associated with stimulus processing involved in WM; this dissociation has also consistently been reported in recent imaging studies with patients^45,46^. Given this functional dissociation, the right aDLPFC may play a key role in RWR via attentional control toward weak representations. Since we excluded any effects directly associated with WM updating, RWR-specific activation in the right aDLPFC would be unrelated to updating weak representations itself. Rather, the right pDLPFC would be associated with the stimulus processing involved in WM in our task.

It could be claimed that some aspects of RWR are similar to refreshing or reflective processes, i.e., redirecting attention toward a specific representation that has recently been presented^47^; in this context, RWR-specific activation could be regarded as refreshing. However, we presume that the right aDLPFC activation is independent of refreshing processes because WM updating, such as 2Back-RP trials in our task, already includes multiple refreshing processes including rehearsal, comparison between items, and updates of representation sets^48^. Moreover, RWR-specific activations were obtained by excluding simple updating in the analyses. In addition, studies on refreshing processes have mainly focused on the refreshing of previously attended items by conceptual definition^49^ and they have reported neural correlates in left-lateralized frontal regions^50^, which differs from our results, i.e., that retrieving previously unattended items recruits the right aDLPFC. Furthermore, refreshing has been suggested to be closely related to searching items activated in WM, which is regarded as a subcomponent for active maintenance of representations within WM^51^.

We note that the other frontal regions found in the current study might be related with functions other than RWR. First, pDLPFC and dmPFC in the right hemisphere were activated by the interaction for RWR contrast, but they overlapped with WM updating-related regions; thus, these regions showed similar activation patterns to simple updating. As previously stated, the right pDLPFC is suggested to be involved in stimulus processing in WM^44^, which seems to be consistent across verbal and nonverbal stimuli^52^. Additionally, the dmPFC was proposed as a core region for WM in a previous meta-analysis^53^, and it has been associated with involvement in monitoring functions in the context of cognitive control^54^. Second, despite being beyond the scope of the current study, our results seem to indicate a relationship between the preSMA and switching from external stimuli to internal representations. Previously, the preSMA has been continuously associated with various types of internally initiated^55^ or covert^56^ responses as well as intentional switching between tasks^57^.

Although this is the first study utilizing fMRI to investigate the neural mechanisms underlying the control processes engaged in retrieving weak representations in WM, our results cannot provide direct evidence of the involvement of these neural mechanisms in creative problem solving. Therefore, future research is warranted to reveal the neural mechanisms of the control processes engaged in RWR during creative problem solving. In conclusion, we have provided the first evidence of neural mechanisms engaged in retrieving weak representations. Our results demonstrate that this process is supported by the right aDLPFC. Furthermore, the current study may provide a new experimental approach to assess the neural and/or cognitive mechanisms of creative problem solving.

## Methods

### Participants

For the current study, 37, young, healthy volunteers were recruited from Kyungpook National University, Daegu, Korea. All participants were right-handed, native Korean speakers with normal or corrected-to-normal vision and without color blindness. None of the participants reported any history of neurological or psychiatric problems. After experimental procedures were explained, participants each provided written informed consent before study participation. After the experiment, all participants were compensated for their participation. Seven participants from the initial sample were excluded due to chance-level performance on one or more experimental conditions; thus, the final sample included 30 participants (15 female, 15 male; age range = 18–33 years, mean = 23.2, SD = 3.3). The current study was approved by the Kyungpook National University Institutional Review Board and conducted in accordance with the guidelines of the Declaration of Helsinki.

### Materials and procedures

#### fMRI experiment

We designed a task that enabled us to measure RWR by comparing retrieval of previously ignored stimuli into WM with that of previously focused upon stimuli; it was based on a task switching paradigm comprising the 2-back updating (2Back) and classification (Classification) tasks (Fig. 1). The stimuli for these tasks consisted of superimposed characters (approximate visual angle: 1°) comprising letters and numbers, which were presented in the middle of a screen on a black background. The letter stimuli included four consonants (D, G, H, and N) and four vowels (A, E, O, and U) while the number stimuli included four even numbers (2, 4, 6, and 8) and four odds (3, 5, 7, and 9). The target was colored red (RGB: 255, 0, 0) or green (RGB: 0, 230, 30) while the distractor was colored gray (RGB: 215, 215, 215); the target and distractor were superimposed onto each other (opacity value: 50%). The red and green stimuli were designated for the Classification and 2Back conditions, respectively, where the color of the target stimulus informed participants of the task being presented. For Classification trials, participants were asked to classify the stimuli as odd/even or vowel/consonant according to whether the current red target was a number or letter, respectively, by pressing a left or right button. For 2Back trials, participants were required to determine whether the current target was identical to the stimulus that appeared two trials before and to press a left or right button for a “yes’ or “no” response, respectively.

The task began with a Classification trial. Specifically, the participants were asked to classify the target stimuli, printed in red, based on their characteristics (i.e., odd/even or vowel/consonant). Subsequently, when the color of the target was changed to green, participants were required to perform the 2Back trials (2Back switch; 2Back-SW). In the 2Back-SW trials, participants were required to identify whether the given stimulus was identical to the one (i.e., a previous red target or gray distractor) presented two trials earlier in the Classification task. 2Back-SW trials were also divided into two types according to whether the current stimulus was presented as a task-relevant target or task-irrelevant distractor in the Classification task: one was the case when the current stimulus was presented as a task-relevant stimulus in the Classification task (2Back-SWr, e.g., the number 2Back-SW trial following successive number Classification trials), and the other was the case when the current stimulus was presented as a task-irrelevant stimulus (2Back-SWi, e.g., the number 2Back-SW trial following consecutive letter Classification trials). After the 2Back-SW trial, 2–4 consecutive 2Back trials were presented within the same target domain; these trials, excluding the trial directly after the 2Back-SW trial, were denoted the 2Back repeat (2Back-RP) trials. The 2Back trial directly following the 2Back-SW trials was treated as a nuisance variable because participants were still required to activate stimuli presented in Classification trials.

After consecutive 2Back trials, the target color was changed to red again and then participants were required to perform the Classification trials (Classification switch; Classification-SW). Classification-SW trials were also divided into two types: where the target domain was relevant to the preceding 2Back trials (Classification-SWr, e.g., the number Classification-SW following number 2Back trials) and where this condition was switched (Classification-SWi, e.g., the number Classification-SW following letter 2Back trials). Subsequently, 2–4 Classification trials (Classification Repeat; Classification-RP) within the same target domain were presented.

Accordingly, 2Back trials were divided into three conditions, namely 2Back-SWr, 2Back-SWi, and 2Back-RP, whereas Classification trials were divided into Classification-SWr, Classification-SWi, and Classification-RP conditions, in which two factors including Task (2Back and Classification) and Switch-type [switch-relevant (SWr), switch-irrelevant (SWi), and repeat (RP)] were fully crossed in a 2 × 3 within-subject design. The experiment consisted of 480 trials divided into four runs. Each of the 2Back-SWi, 2Back-SWr, Classification-SWi, and Classification-SWr conditions included 30 trials, while the 2Back-RP and Classification-RP conditions included 120 and 180 trials, respectively. Each run began with a central fixation and followed an additional Classification trial; these were excluded from the task condition. An event-related design was employed in which the stimuli were presented for 500 ms with intertrial intervals (ITIs) of 2000–4000 ms (mean: 3000 ms; increased by 500 ms). Participants were instructed to respond to the task as quickly and accurately as possible with button presses using their left or right thumbs. Task programming and stimulus presentation were conducted via E-Prime 2.0.

#### Assessment of creativity and attention-demanding task performances

Participants were administrated AUT as a creativity task as well as arrow flanker and response switching tasks as attention-demanding tasks outside of the MRI room. These were presented in a counterbalanced manner across participants; each half of the participants performed these tasks before or after MRI scanning. For AUT, participants were instructed to generate appropriate alternative uses for three common objects (“Brick,” “Key,” and “Newspaper”); they recorded as many uses as possible for each item within 2 min using a paper and pen. To improve the reliability and representativeness of the AUT scores, data from a sample of 97 participants who participated in pilot behavioral and fMRI experiments were used to calculate the AUT scores. Among them, 60 participants took part in a pilot experiment to identify the optimal task configurations for fMRI experiments, including stimulus duration, inter-trial interval, and task difficulty. The fluency score was calculated by counting the number of correct solutions. The flexibility score was computed by counting the number of different solution categories. The originality score for each response was computed by dividing the percentage of the given response over the set of entire responses. The different points were then assigned to individual responses according to their percentages (< 1%: 2 points; < 5%: 1 point; and ≥ 5%: 0 points). The points for each of the three objects were summed and divided by the number of correct responses^58^. In addition to the three subscores, a total score was used for a representative single index of creativity task performance^59,60^. To compute the total score, principal component analysis (PCA) was conducted for 97 participants from the current fMRI study, as well as a pilot behavioral experiment (see, Supplementary Materials), before extracting the AUT scores for 30 participants from the current study.

For the arrow flanker task, a horizontal array of five white arrows in the center of the screen was presented on a black background. The middle arrow was the target while the others were non-target distractors. The arrows either pointed in the same direction (congruent trials: “< < < < <” or “> > > > >”) or opposite direction (incongruent trials: “< < > < <” or “> > < > >”). The task required participants to respond to the direction of the target with a left or right button press as quickly and accurately as possible while ignoring distractors. It consisted of 128 trials (64 congruent and 64 incongruent trials). The stimuli were presented for 500 ms with a fixation cross presented as an ITI in the middle of the screen for 2000 ms. The interference effect, which represents an individual’s ability to focus their attention on an external stimulus^61^, was measured by subtracting the mean retention times (RTs) of congruent trials from those of incongruent trials.

Stimuli for the response switching task consisted of four even numbers (2, 4, 6, and 8) and four odd (3, 5, 7, and 9), which were colored either green or red. Participants were asked to classify the targets as odd or even by pressing their left or right buttons, respectively, when the target color was green; their responses were then reversed when the target was red. The task included 40 switch trials and 120 repeat trials. The stimuli were presented for 500 ms with a fixation cross presented as an ITI in the middle of the screen for 2000 ms. The switch cost, which indicates individual differences in behavioral flexibility^62^, was measured by subtracting the mean RTs of repeat trials from those of switch trials.

### Imaging acquisition

fMRI images were acquired using a 3 T Siemens Magnetom Skyra system equipped with a 20-channel head coil (Medical Device Development Center at Daegu-Gyeongbuk Medical Cluster). Task stimuli were presented via MRI-compatible goggles (NordicNeuroLab Visual System, Bergen, Norway; resolution: 800 × 600; refresh rate: 60 Hz) mounted on the head coil. Functional images were collected using a T2*-weighted gradient echo planner image (EPI) sequence [repetition time (TR): 2,000 ms; echo time (TE): 35 ms; flip angle (FA): 80°; field of view (FOV): 224 mm^2^; 33 interleaved slices; voxel size: 3.5 mm^3^; and 217 volumes per run]. Three dummy images preceded each run to allow for magnetic stabilization and these were discarded prior to image processing. High-resolution T1-weighted magnetization-prepared rapid gradient-echo (MPRAGE) images were also acquired (TR: 2,530 ms; TE: 3.44 ms; FA: 9°; FOV: 256 mm; voxel size: 1 mm^3^).

### Image preprocessing and voxel-wise analyses

Image preprocessing was conducted using standard procedures: after discarding the first three functional volumes, the temporal disparity between slices was corrected by sinc interpolation^63^ and motion artifacts due to head motion were minimized by realigning the timing-corrected images to the first image of the first run with a six-parameter rigid body spatial transformation^64,65^. These images were coregistered onto the MPRAGE image for each subject and normalized into the International Consortium for Brain Mapping (ICBM) 152 template (2 mm isotropic voxels) using unified segmentation-based normalization with a 12-parameter affine and non-linear transformation^66^. These images were then resampled to 2 mm isotropic voxels and spatially smoothed by a 6-mm full-width/half-maximum (FWHM) Gaussian kernel. Finally, highpass filtering with a 128 s cutoff was applied to the images to eliminate low-frequency drifts.

Statistical analyses at the subject-level were conducted via a general linear model (GLM) using a canonical hemodynamic response function with temporal and dispersion derivatives. The model design matrix for each subject included the regressors for each run of 2Back-SWi, 2Back-SWr, 2Back-RP, Classification-SWi, Classification-SWr, and Classification-RP. In addition to these regressors-of-interest, the 2Back trials that immediately followed the 2Back-SWi and Classification-SWr trials, the first trial of each run and error trials, and the six head motion parameters derived from realignment correction were entered into the design matrix as regressors-of-no-interest. The regressors were fitted to the fMRI data to produce voxel-wise beta estimates for each condition. From the subject-level model, individual contrast images were generated for six experimental conditions, including 2Back-SWi, 2Back-SWr, 2Back-RP, Classification-SWi, Classification-SWr, and Classification-RP.

For the second-level group analyses, individual contrast images corresponding to the six experimental conditions from the subject-level analyses were entered into a random effects model^67^ with a 2 (Task: 2Back & Classification) × 3 (Switch-type: SWr, SWi & RP) repeated measures ANOVA using a flexible factorial analysis in SPM. The accuracy of each condition was also entered as a covariate to control for differences in task difficulty between the conditions. For all statistical analyses, the statistical threshold was set at *p* < 0.05 and corrected for multiple comparisons using false discovery rate at the cluster level, with an underlying voxel level of uncorrected *p* < 0.001^68,69^.

Whole-brain analyses were performed to identify brain regions associated with RWR via an interaction contrast, [(2Back-SWi–2Back-SWr) > (Classification-SWi–Classification-SWr)], which represented greater activations of task-irrelevant internal representations (i.e., 2Back-SWi–2Back-SWr) compared to task-irrelevant external stimuli (i.e., Classification-SWi–Classification-SWr). Because the regions activated by the interaction contrast could involve greater loads of WM updating or switching, it was important to distinguish RWR-specific regions from any regions commonly involved in simple updating and switching, irrespective of RWR contrast. Accordingly, regions associated with the simple effects of WM updating, [2Back-RP > Classification-RP], and switching for the 2Back condition, [(2Back-SWi + 2Back-SWr)/2 > 2Back-RP)], were specified, and these regions were then exclusively masked for the regions activated by RWR contrast. To determine the minimum cluster size for the cluster level correction (*p* < 0.05), statistical significance was calculated using Monte Carlo simulations (10,000 iterations) implemented in the AFNI 3dClustSim tool (https://afni.nimh.nih.gov/pub/dist/doc/program_help/3dClustSim.html). Consequently, clusters with a minimum of 73 contiguous voxels (*p* < 0.001 at the voxel level) were considered to be significant (*p* < 0.05).

An additional interaction was tested to differentiate RWR-specific regions from those associated with switching from Classification to 2Back trials: switching from external stimuli to internal representations regardless of their intensities was tested via a 2 × 3 interaction (i.e., [(2Back-SWi + 2Back-SWr)/2 − 2Back-RP)] > [(Classification-SWi + Classification-SWr)/2 − Classification-RP]).

### Region-wise analyses

Regions of interest (ROI) were defined as 3-mm radial spheres centered on the peak coordinates of the clusters, which were identified by the interaction contrast. For each participant, percent signal changes (PSCs) corresponding to each experimental condition were extracted from the ROI using the Marsbar toolbox (http://marsbar.sourceforge.net/). The functional ROIs were labeled based on a parcellation into areas that have been functionally defined in other imaging studies by using Neurosynth^70^ (https://neurosynth.org/). ROI analyses were performed to characterize brain activation patterns and to describe relationships between the neural activations and behavioral measurements of creativity task performance (i.e., the AUT scores) as well as attention-demanding task performance (i.e., the interference effect and switch cost).

Accordingly, for the ROI identified by the RWR interaction contrast (namely, the significant interaction), PSCs were tested using pairwise comparisons between 2Back-SWi and 2Back-SWr, and between Classification-SWi and Classification-SWr, using paired samples t-tests, to identify the source of interaction. Similarly, the activation patterns of the ROI identified via the 2 × 3 interaction were examined using pairwise comparisons of PSCs between 2Back-SW and 2Back-RP and between 2Back-SWi and 2Back-SWr, and then between Classification-SW and Classification-RP and between Classification-SWi and Classification-SWr. Subsequently, correlation analyses were conducted between the PSCs of these regions and the aforementioned behavioral measurements. Among the ROIs identified by the RWR contrast, the ROIs in which interaction was driven by the increased activity of 2Back-SWi relative to 2Back-SWr, rather than the other conditions, were selected. For these ROIs, the differences in PSCs between 2Back-SWi and 2Back-SWr were calculated, and these differences were correlated with the AUT scores, interference effect, and switch cost. In addition, for the ROI identified by the 2 × 3 interaction, the neural 2Back switch cost (i.e., 2Back-SW–2Back-RP) and Classification switch cost (i.e., Classification-SW–Classification-RP) were calculated and correlated with the behavioral measurements. The significance level of the correlation results was corrected at *p* < 0.05 (equivalent to uncorrected *p* < 0.0125) for multiple comparisons using Bonferroni correction.

## Supplementary Information

Supplementary Information.

## Data Availability

We have deposited behavioral and PSCs data used for the analyses in the Open Science Framework repository at https://osf.io/7xprg/ and fMRI maps of all contrasts depicted in the manuscript on NeuroVault at https://identifiers.org/neurovault.collection:8715.
