# Supplementary material for: The role of the right prefrontal cortex in the retrieval of weak representations
Source: Sci Rep. 2022 Mar 16;12:4537. doi: 10.1038/s41598-022-08493-6 (PMC8927597; doi:10.1038/s41598-022-08493-6)
Supplement: Supplementary file 1 — Supplementary Information. [file 41598_2022_8493_MOESM1_ESM.docx]

**Supplementary Materials**

**The role of the right prefrontal cortex in retrieval of weak representations**

Kyongmyon Yi*^a^*, Juyeon Heo*^a^*, Jiyun Hong*^a^*, and Chobok Kim*^a*^*

*^a^ Department of Psychology, Kyungpook National University, Daegu, 41566, South Korea*

*Corresponding author:

Chobok Kim, Ph.D.

Associate Professor

Department of Psychology

Kyungpook National University

Daegu, 41566

South Korea

Email: [ckim@knu.ac.kr](mailto:ckim@knu.ac.kr)

Phone: +82-53-950-5250

Fax: +82-53-950-5243

| 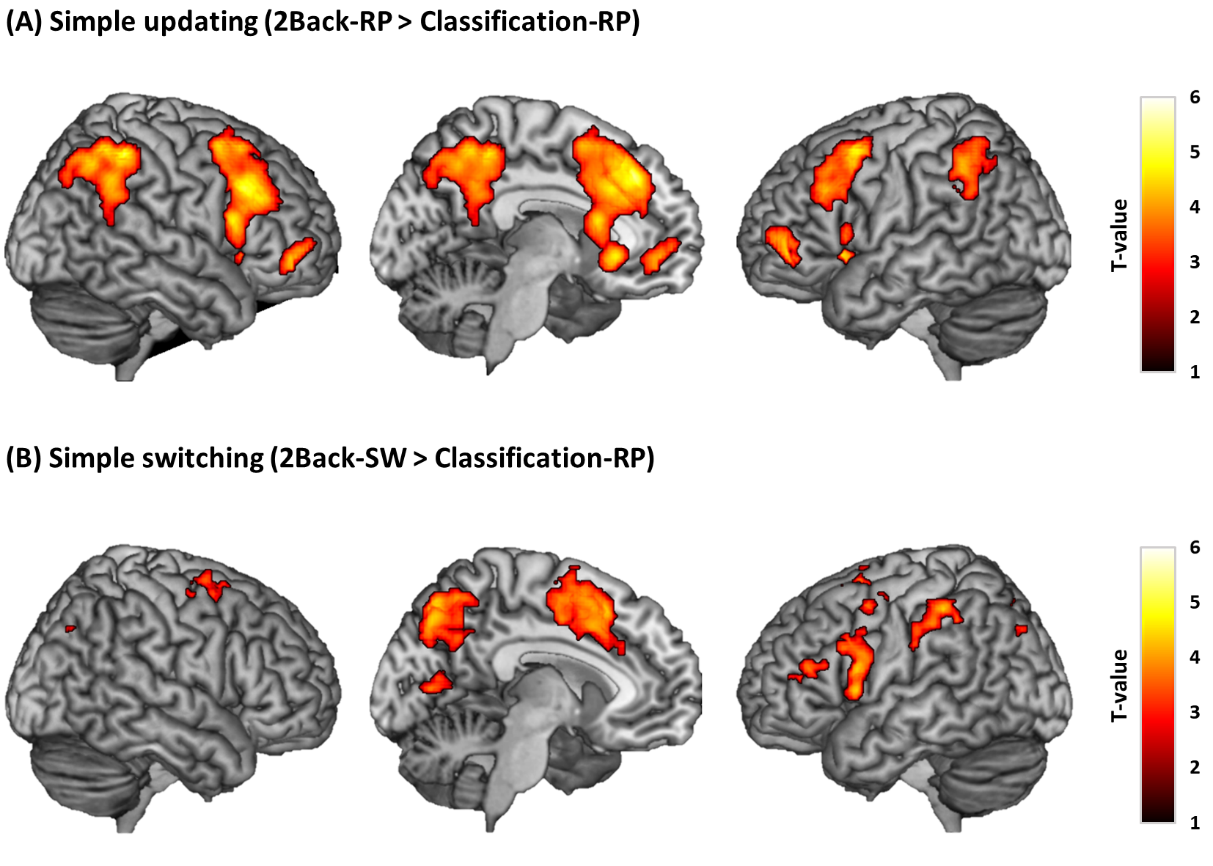 |
| --- |
| Fig. S1. Brain activation maps associated with the simple effects of (A) WM updating, i.e., 2Back-RP > Classification-RP and (B) switching, i.e., 2Back-SW > 2Back-RP. The statistical threshold maps were shown at *p* < 0.05 cluster-level correction for multiple comparisons with the cluster size defined at a voxel level, uncorrected *p* < 0.001. Color bars represent the t-values. The activated clusters are overlapped onto the ch2better.nii template using mricron software (version 09.02.2019, https://www.nitrc.org/projects/mricron)^1^. |

| 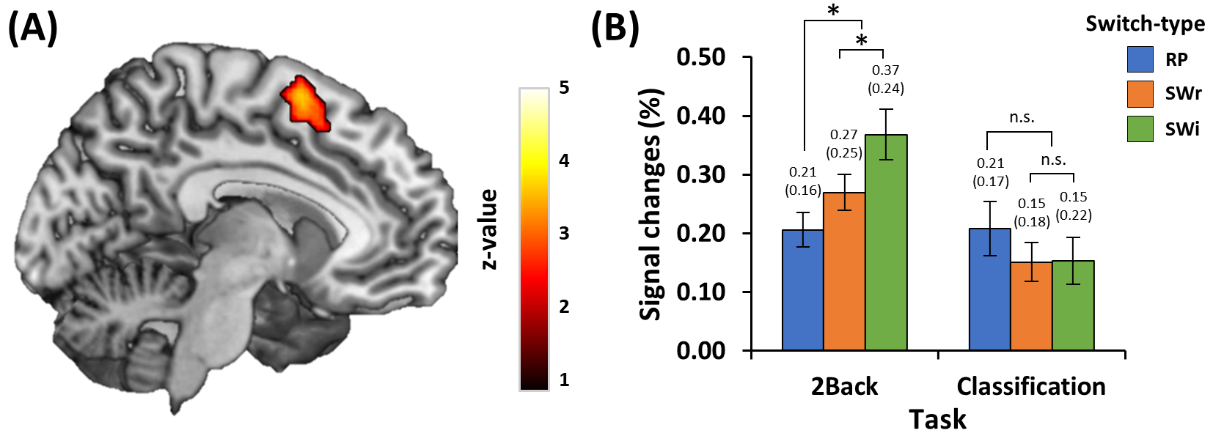 |
| --- |
| Fig. S2. (A) Activation of the presupplementary motor area (preSMA) observed in a 2 × 3 interaction (peak MNI: x = −4, y = 10, z = 62; cluster size: 226; z-value = 4.94). (B) Percent signal changes for each task condition. The *post-hoc* pairwise comparisons were tested using paired simples t-tests based on significant interactions. The statistical threshold maps were shown at *p* < 0.05 cluster-level correction for multiple comparisons with the cluster size defined at a voxel level, uncorrected *p* < 0.001. Color bars represent the t-values. The activated clusters are overlapped onto the ch2better.nii template using mricron software (version 09.02.2019, https://www.nitrc.org/projects/mricron)^1^. |

| Table S1. Comparisons between two groups (Pilot and fMRI) across AUT subscores and the total score. | | | | | | |
| --- | --- | --- | --- | --- | --- | --- |
|  | | N | M | SD | t | p |
| Fluency | Pilot | 60 | 3.62 | 1.76 | −1.708 | 0.091 |
|  | fMRI | 37 | 4.25 | 1.77 |  |  |
| Flexibility | Pilot | 60 | 3.39 | 1.57 | −1.698 | 0.093 |
|  | fMRI | 37 | 3.95 | 1.57 |  |  |
| Originality | Pilot | 60 | 0.59 | 0.26 | 1.408 | 0.162 |
|  | fMRI | 37 | 0.51 | 0.22 |  |  |
| Total score | Pilot | 60 | −0.11 | 0.99 | −1.404 | 0.163 |
|  | fMRI | 37 | 0.18 | 1.00 |  |  |

**References**

1 Rorden, C., Karnath, H.-O. & Bonilha, L. Improving Lesion-Symptom Mapping. *J. Cogn. Neurosci.* **19**, 1081-1088 (2007).
